# Editorial: Genetic mechanisms in diabetes pathogenesis

**DOI:** 10.3389/fendo.2026.1896584

**Published:** 2026-06-15

**Authors:** Roma Patel, Nirali Rathwa, Dhanakesaran Bodhini

**Affiliations:** 1 Dominick P. Purpura Department of Neuroscience, Albert Einstein College of Medicine, Bronx, NY, United States; 2 Programming & Corporate Innovation Division, MaRS Discovery District, Toronto, ON, Canada; 3 Department of Molecular Genetics, Madras Diabetes Research Foundation (ICMR Collaborating Centre of Excellence), Chennai, TN, India

**Keywords:** beta cell dysfunction, diabetes mellitus Type 2, diabetic complications, epigenetic regulation, genetic heterogeneity, MODY (mature onset diabetes of the young), non-coding RNAs

Diabetes mellitus is increasingly understood not as a single disease, but as a group of biologically distinct disorders that converge on similar clinical outcomes. Patients with comparable glucose levels or HbA1c values may differ substantially in the molecular pathways driving disease progression, including β-cell dysfunction, adipose tissue abnormalities, immune dysregulation, oxidative stress, epigenetic regulation, or insulin receptor defects. This growing recognition of disease heterogeneity is reshaping diabetes research and shifting the field toward mechanism-based classification and precision medicine approaches. The studies collected in this Research Topic reflect these advances and together provide important insights into the genetic, epigenetic, transcriptomic, inflammatory, and metabolic pathways contributing to diabetes and its complications. An important strength of this Research Topic is its broad international representation: the studies span populations from India, Sri Lanka, Pakistan, China, Qatar, Russia, Brazil, and Latin America, emphasizing the importance of ancestry-specific investigations and the growing global reach of diabetes genetics research.

## Immune and epigenetic regulation in T2D

A major theme across this Research Topic is the contribution of immune signaling, inflammation, and epigenetic regulation to type 2 diabetes (T2D) pathogenesis, as mechanisms that operate alongside inherited genetic variation to modulate disease susceptibility and progression. Chen et al. combined transcriptomic, methylomic, and proteomic datasets using summary data-based Mendelian randomization to identify immune-regulatory pathways associated with T2D, highlighting *LTBP3*, *PLXNB2*, and *OPRL1* as genes linking immune signaling, gut physiology, and metabolic dysfunction. Experimental work in diabetic mouse models suggested that electroacupuncture may partially restore intestinal physiology and alter expression of these pathways, though these findings remain preliminary and require further validation in humans. At the post-transcriptional level, Yin et al. reviewed the expanding role of N6-methyladenosine (m6A) RNA modification in diabetes and its complications, describing m6A as a dynamic and reversible regulatory mechanism affecting β-cell function, insulin secretion, inflammation, and vascular injury across multiple tissues. Regulators such as *METTL3*, *METTL14*, *FTO*, *WTAP*, and *YTH* domain proteins were shown to exert tissue-specific and context-dependent effects, sometimes opposing each other within the same disease context, underscoring the complexity of post-transcriptional regulation in metabolic disease. Ling et al. provided a focused mini−review on β-cell vulnerability from a different angle, reviewing how oxidative stress-induced protein carbonylation impairs β-cell survival and insulin secretion, and positioning *NRF2*-mediated antioxidant pathways as candidate therapeutic targets. These mechanistic layers illustrate that diabetes progression cannot be reduced to inherited sequence variation alone. Waqar et al. reported significant associations between *TNF-α* promoter polymorphisms rs1800629 and rs361525 and T2D susceptibility in a Punjabi population from Pakistan, supporting the role of chronic low-grade inflammation in disease risk, consistent with prior work linking *TNF-α* genetic variants to dyslipidemia and T2D susceptibility in South Asian populations (1). Similarly, Parmar et al. showed that the *LEPR* rs1137101 polymorphism, alongside altered leptin and soluble leptin receptor levels, was associated with T2D susceptibility in the Gujarat population, reinforcing the importance of adipokine signaling and leptin resistance as heritable components of metabolic dysfunction.

## Non-coding RNAs and diabetic complications

The overlap between metabolic and infectious disease susceptibility has gained renewed scientific attention, and Chávez-Domínguez et al. offer a compelling illustration of this. Their systematic review identified shared microRNA signatures, including miR-21, miR-155, and miR-223, that have been implicated in both T2D and tuberculosis susceptibility through dysregulated inflammatory, apoptotic, and glucose metabolic pathways. This work highlights the immunometabolic cross-talk that may help explain the well-established but incompletely understood heightened susceptibility to tuberculosis in people with diabetes, a comorbidity that has been quantified as approximately a three-fold increase in TB risk among diabetic individuals (2). Palihaderu et al., working in a Sri Lankan T2D cohort, identified circulating miR-29a-3p and miR-375-3p as significantly dysregulated in diabetic compared with normoglycemic individuals, linking these miRNAs to β-cell dysfunction and insulin signaling pathways and supporting their potential as minimally invasive biomarkers, while appropriately acknowledging the need for larger-scale validation. Extending these transcriptomic investigations into end-organ complications, Zhang and Yang identified *RPL11* as a potential biomarker associated with diabetic retinopathy progression and proposed interactions with bisphenol A exposure, raising questions about how environmental factors may intersect with molecular disease pathways in diabetic tissues. In diabetic nephropathy, Zhang et al. demonstrated that miR-216a-5p protects against high glucose-induced mesangial cell injury by suppressing HMGB1/RAGE signaling and thereby reducing inflammation, fibrosis, and apoptosis. These studies demonstrate how transcriptomic and microRNA-based approaches are contributing both to biomarker discovery and to mechanistic understanding of diabetic complications, though a consistent gap remains the transition from discovery-stage findings in small cohorts to validated clinical tools.

## Adipose and pancreatic microenvironment dysfunction

The field has moved well beyond a pancreas-centric view of diabetes pathogenesis, and several contributions in this Research Topic make that case forcefully. Saxena et al. demonstrated that altered expression of lipodystrophy-associated genes across visceral and subcutaneous adipose depots strongly correlated with insulin resistance, glycemia, and metabolic syndrome severity in Asian Indians. Their findings support the concept of “adiposopathy,” in which dysfunctional adipose tissue acts as a primary driver of metabolic disease, and are particularly relevant to the South Asian “thin-fat” phenotype, where insulin resistance and complications emerge at BMI thresholds lower than those typical of European populations. Notably, a relatively small oligogenic gene subset was sufficient to stratify metabolic syndrome severity, pointing to a tractable entry point for early risk prediction. This is consistent with evidence from data-driven clustering in Asian Indians showing that distinct T2D subgroups carry different risks of microvascular complications, further underscoring the clinical relevance of phenotypic heterogeneity within South Asian populations (3). Adding granularity to adipose biology, Badran et al. compared abdominal and femoral subcutaneous depots in non-obese insulin-resistant individuals and identified distinct transcriptional regulation of *GATA3* and *PPAR-γ* between compartments, with the femoral depot showing unique responses potentially linked to vitamin D status. Though limited by sample size, the findings reinforce that fat distribution carries mechanistic information that extends beyond fat mass alone.

Turning to the pancreatic microenvironment, Qiu et al. identified a T2D-specific pancreatic stellate cell signature characterized by high expression of *COL1A2*, *VCAN*, and *SULF1*, using integrated single-cell and bulk transcriptomic analyses. This signature was enriched in T2D but absent in type 1 diabetes islets, a distinction pointing to disease-specific stromal remodeling rather than a generalized injury response, and was linked to fibrosis-driven β-cell dysfunction. The identification of interacting long non-coding RNAs, including *LINC00472*, *SNHG14*, and *ZFAS1*, further suggests a regulatory axis that may interface peripheral immune signals with the islet microenvironment, expanding the landscape of potential therapeutic targets in T2D well beyond the β-cell itself.

## Ancestry-specific genetic architecture and gestational diabetes

One of the most consistent messages across this Research Topic is that T2D is not one disease even at the population level. Markelova et al. make this point with particular clarity, applying partitioned polygenic score analyses across three Russian ancestry groups to show that the dominant biological drivers of T2D differ substantially by genetic background. Yakut populations exhibited stronger genetic signatures for β-cell dysfunction and hyperinsulinemia with lower obesity-related burden, mirroring East Asian metabolic patterns, while Chechen and Tatar groups showed more obesity-driven mechanisms accompanied by distinct anthropometric and lipid profiles. Strikingly, the β-cell dysfunction and hyperinsulinemia signals in Yakuts appeared genetically independent of each other, suggesting these are not simply two aspects of the same process but mechanistically separable contributions that ancestry helps unmask. These findings also carry practical implications, as polygenic risk tools calibrated primarily on European cohorts may misrepresent disease risk in populations with distinct underlying pathogenic pathways, a well-documented limitation of current PRS methodology that underscores the urgent need for more ancestrally diverse genomic studies (4). Evidence from South Asian and European population comparisons further confirms that even the genetic determinants of age at diabetes diagnosis differ between ancestries, reinforcing the need for ancestry-specific genomic investigations (5).

House et al. extend this argument to glycation biology, identifying novel genetic determinants of the hemoglobin glycation index (HGI) in the ACCORD trial that are largely independent of known HbA1c and glycemic loci. Sex-specific signals, including *GALNT11* in women and *HECW2* in men, alongside ancestry-specific associations such as *WHRN* in Black participants and *USF1/NXNL2* in Hispanic individuals, reinforce that even well-established glycemic biomarkers harbor biological complexity that standard population-level analyses can obscure.

Gestational diabetes mellitus (GDM) studies in this Research Topic further highlight the value of population-specific and cross-ancestry investigation. Zhang et al. identified associations between *MIF* rs1007888, ARAP1 rs1552224, and GDM susceptibility in Chinese women, with MIF increasing risk and ARAP1 showing a protective association in specific subgroups (for e.g. younger women and those with higher BMI), while Zhao et al. conducted a cross-ancestry genome-wide association meta-analysis across British, Finnish, and Chinese populations, identifying novel susceptibility genes *ELL2* and *ATRAID* alongside pathways related to regulation of insulin secretion, regulation of protein secretion, and negative regulation of hexokinase activity. GDM genetic studies have historically been constrained to single-population designs; the cross-ancestry approach adopted by Zhao et al. improves both discovery power and generalizability of findings, an important methodological step for a condition that disproportionately affects women from diverse ethnic backgrounds.

## Monogenic diabetes and rare genetic variants

Monogenic diabetes is frequently misclassified as type 1 or type 2, and that misclassification has real treatment consequences. Several studies in this Research Topic demonstrate how comprehensive genetic diagnosis corrects that error and directly improves patient outcomes. This is particularly relevant in South Asian contexts, where the profile and frequency of monogenic diabetes subtypes differ substantially from European populations, as demonstrated by a recent pan-India registry study documenting significant clinical and genetic heterogeneity across multiple MODY subtypes (6). Sechko et al. characterized *HNF4A*-MODY in Russian pediatric patients, showing that genetic confirmation facilitated insulin-to-sulfonylurea transition in 43% of cases. Wang et al. demonstrated that mutations in distinct *HNF1A* functional domains carry different metabolic consequences, as DNA-binding domain mutations were associated with earlier disease onset and altered lipid profiles, establishing genotype-informed prognostication as a realistic clinical tool. That diagnostic precision depends, however, on testing strategies that go beyond standard sequencing. Lei et al. identified a heterozygous deletion spanning *HNF1A* exons 1-10, functionally amounting to a whole-gene-loss-of-function event, in a Chinese MODY family that was invisible to next-generation sequencing but confirmed by copy number variant analysis, and which enabled the same insulin-to-sulfonylurea transition seen in the HNF4A cohort. Gong et al. showed that a non-canonical *HNF1B* splice-site variant caused abnormal mRNA splicing and protein truncation, with pathogenicity established only through minigene functional assays. These two studies together argue that copy number analysis, splice-site assessment, and functional validation are no longer optional additions to monogenic diabetes diagnostics but integral to accurate genetic diagnosis.

Several reports in this section also broaden the recognized phenotypic boundaries of known monogenic conditions. Shao and Xu described a ketosis-prone presentation of MODY6 caused by a *NEUROD1* 5’ UTR mutation, a phenotype traditionally considered inconsistent with this subtype, in a single-family patient who achieved stable glycemic control after insulin discontinuation with dipeptidyl peptidase-4 (DPP-4) inhibitor therapy, suggesting preserved incretin responsiveness. Wei et al. reported severe insulin resistance in a patient with autoantibody-negative type 1 diabetes carrying an *IGF2BP2* variant classically associated with T2D susceptibility, illustrating how such polygenic background may contribute to marked insulin resistance across diagnostic categories. Marcelino do Nascimento et al. characterized pathogenic *INSR* variants across five unrelated families presenting with hyperinsulinemic hypoglycemia and insulin resistance, revealing a distinctive metabolic signature of normal BMI, severe hyperinsulinemia, paradoxical ketonemia, and postprandial hypoglycemia, and presenting findings that are consistent with a semidominant inheritance model that accommodates the broad phenotypic spectrum observed. Perhaps the most thought-provoking contribution in this section is the CRISPR-Cas9 *PDX1*-P33T mouse model developed by Harten et al. Despite substantial prior evidence supporting the pathogenicity of this MODY4-associated mutation, homozygous mutant mice failed to reproduce the expected diabetic phenotype under standard conditions and instead exhibited transcriptomic evidence of adaptive stress responses that eventually broke down under high-fat feeding. The divergence between murine and human outcomes is a timely reminder that genotype-phenotype relationships are shaped by species-specific biology, and that negative results in animal models can still provide meaningful biological insight.

## Conclusion

With 589 million adults currently living with diabetes globally, a figure projected to reach 853 million by 2050 (7), the studies presented in this Research Topic reinforce a view of diabetes as a condition shaped by the intersection of inherited genetic variation, post-transcriptional and epigenetic regulation, immune and inflammatory signaling, tissue-specific dysfunction, environmental exposure, and ancestry-driven biological architecture. The value of this Research Topic lies not in any single finding but in the cumulative argument it assembles: that similar clinical phenotypes can arise from fundamentally different molecular mechanisms, and that distinguishing between those mechanisms, through more comprehensive genetic testing, functional validation, multi-omics integration, and ancestry-aware analysis, has direct consequences for how patients are diagnosed and treated. The shift from phenotype-based toward mechanism-based classification is already underway, and the studies here accelerate it.

As diabetes prevalence continues to rise globally, building diverse, well-phenotyped cohorts that reflect the full range of populations affected by the disease will only become more important. Many of the molecular insights described in this Research Topic, particularly in non-coding RNA biology and epitranscriptomics, still await validation in large human datasets. Closing that gap will require sustained international collaboration and investment in genomic infrastructure in regions historically underrepresented in diabetes research. We hope this Research Topic encourages precisely those efforts, and that the findings presented here contribute to more individualized, mechanism-informed approaches to diabetes prevention, diagnosis, and treatment.
